# Screening of variables affecting the selective leaching of valuable metals from waste motherboards’ PCBs

**DOI:** 10.1007/s11356-024-32793-1

**Published:** 2024-03-09

**Authors:** Vahid Rahimi, Emilio Antonio Inzulza-Moraga, Diego Gómez-Díaz, María Sonia Freire, Julia González-Álvarez

**Affiliations:** Department of Chemical Engineering, School of Engineering, Universidade de Santiago de Compostela, Rúa Lope Gómez de Marzoa s/n, 15782 Santiago de Compostela, Spain

**Keywords:** Waste motherboards’ PCBs, Leaching, Base metals, Precious metals, Screening design

## Abstract

The presence of valuable and hazardous metals in waste printed circuit boards, especially, motherboards, makes their recovery necessary as implies great economic and environmental advantages and develops urban mining processes. Hence, this research is focused on the selective leaching of Cu, Pb, and Sn as base metals using nitric acid and hydrochloric acid and Au, Ag, and Pd as precious metals using thiourea and sodium thiosulfate from waste motherboards’ PCBs in a sequential eco-friendly two-stage process. Previously, thiourea and sodium thiosulfate were used as leaching agents to investigate their applicability for the leaching of metals from PCBs in a single-stage process. Screening experimental design was applied to screen the variables affecting the leaching process in order to evaluate their impact on the recovery of metals and select the significant factors. The results demonstrated that base and precious metals can be leached appropriately in two consecutive stages compared to a single-stage process. Nitric acid was found to be a much more efficient agent to leach Cu and Pb in comparison with hydrochloric acid which was more suitable for the leaching of Sn. In the case of precious metals, higher amounts of Au were leached using thiourea, whereas sodium thiosulfate was able to leach more Pd. Roughly similar results were obtained for the leaching of Ag using these leaching agents. Nitric acid concentration, average particle size, temperature, and leaching time were found to be significant to maximize the leaching of Cu and Pb and minimize that for Au, Ag, and Pd in the first stage. Initial pH was the only variable influencing the second stage, in particular, Au leaching by thiourea.

**Supplementary Information:**

The online version contains supplementary material available at 10.1007/s11356-024-32793-1.

## Introduction

The rapid growth in the advancement of technology and the electronic industry has led to a significant increase in the amount of electronic waste (e-waste). E-waste generally refers to all kinds of electronic equipment and devices which are discarded and reached the end of their useful life (Yaazhmozhi et al. 2020). Printed circuit boards (PCBs) are the main parts of e-waste which are mostly contained in personal computers, televisions, mobile phones, and other information technology (IT) and telecommunications equipment accounting for an estimated 3–5% of e-waste (Hao et al. 2020). Significant amounts of waste PCBs (roughly 1.5 million tons) are discarded yearly (Kaya 2016). Waste motherboards’ PCBs obtained from obsolete computers can be considered as a kind of “urban mine” or valuable secondary resources including base (such as Cu, Pb, and Sn) and precious metals (such as Au, Ag, and Pd). Moreover, most of these waste PCBs are incinerated or transferred to the landfill resulting in environmental and health problems owing to the production of toxic compounds such as furans and dioxins and leaching of some heavy metals such as lead into the landfill sites (Jadhav and Hocheng 2015; Rocchetti et al. 2013). Therefore, recycling the waste motherboards’ PCBs is required to recover valuable metallic resources and protect the environment at the same time.

Though the primary goal is to recover valuable metals from e-waste, particularly precious metals (Au, Ag, and Pd), due to changes in manufacturing processes leading to a decrease in their amount in PCBs, the interest is also addressed to the recovery of base metals such as Cu, Pb, and Sn, which are found in substantial concentrations in PCBs. (Castro and Martins 2009; Fogarasi et al. 2014). Moreover, base metal recovery from waste PCBs has great environmental profit. For instance, lead which is used together with tin for soldering electronic components on the surface of motherboards’ PCBs has detrimental effects on human health and the environment when it encounters in liquid waste streams created by industrial activities since these hazardous liquid solutions can be absorbed by the soil and pollute the water (Parvez et al. 2021), so its recovery and that of other base metals are of great interest. In addition, the selective leaching of base metals in a preliminary step can lead to significantly improve the subsequent recovery of precious metals (Sheng and Etsell 2007; Birloaga and Vegliò 2016; Birloaga et al. 2014).

Among the recovery processes of metals from waste PCBs including hydrometallurgy, pyrometallurgy, and biometallurgy, the first one has attracted extensive attention due to its low environmental impact, low operational cost, and ease of operation (Cui and Anderson 2016; Birloaga and Veglio 2018; Tuncuk et al. 2012). This process consists of a pre-treatment of waste PCBs followed by leaching, and after that, several separation operations could be applied in order to recover the metals. Leaching as the first stage of hydrometallurgical process using cyanide and acids such as sulfuric acid (H_2_SO_4_), hydrochloric acid (HCl), and nitric acid (HNO_3_) has been extensively applied owing to its simple process and low cost. Cyanide is an efficient lixiviant for the recovery of precious metals such as Au (Işıldar et al. 2018) whereas base metals such as Cu and Sn could be recovered from waste PCBs using HNO_3_, HCl, and H_2_SO_4_ (Chaurasia et al. 2013). Hao et al. (2022) used H_2_SO_4_ together with H_2_O_2_ to recover Cu from waste mobile phones’ PCBs. However, the poor solubility of Cu in H_2_SO_4_ due to the weak oxidation of the acid and the necessity of employing an additional oxidant such as hydrogen peroxide (H_2_O_2_) to improve Cu leaching has restricted its application (Silvas et al. 2015; Torres and Lapidus 2016). Fogarasi et al. (2019) developed a process for the leaching of Pb and Sn from waste solder alloy using H_2_SO_4_ after eliminating remnants of copper and iron. HCl is normally used along with oxidants such as chlorine (Cl_2_), H_2_O_2_ or HNO_3_ as an oxidizing acid for the recovery of base and precious metals (Cui and Anderson 2016; Bas et al. 2014). Jadhav and Hocheng (2015) found that HCl possesses a high potential as leaching agent working on the recovery of metals from large PCBs in comparison with other different leaching agents such as HNO_3_, H_2_SO_4_, and C_6_H_8_O_7_ (citric acid). Kim et al. (2011) reported a process to recover Cu and Au from waste mobile phones’ PCBs using HCl in combination with Cl_2_. Imre-Lucaci et al. (2017) applied HCl together with H_2_O_2_ as an oxidant to recover Au from waste PCBs. Rao et al. (2021) used HNO_3_ to leach Cu and Ni from obsolete mobile phones’ PCBs. Vlasopoulos et al. (2023) used *aqua regia* for the leaching of Au from waste-printed circuit boards after recovery of base metals and silver in sequential stages.

However, despite the high potential of cyanide and aqua regia for the leaching of precious metals, their toxicity and corrosive problems limit their use and research has focused on the application of alternative agents such as thiourea and thiosulfate for the leaching of precious metals such as Au and Ag. For instance, Camelino et al. (2015) reported a process for the leaching of Au from waste cell phones’ PCBs using thiourea and ammonium thiosulfate after preliminary extraction of Cu. Alvarado-Macias et al. (2016) discovered that sodium thiosulfate has a high potential to leach Ag. Jing-Ying et al. (2012) applied thiourea together with an oxidant such as ferric ion (Fe^3+^) to extract Au and Ag from waste mobile phones' PCBs.

On the other hand, as mentioned before, prior research has mostly concentrated on the leaching of metals from waste PCBs obtained from discarded mobile phones. Therefore, the sequential leaching of base and precious metals from PCBs, especially motherboards with lower concentration of precious metals compared to mobile phones, can be a challenge. Thus, in this work, first, thiourea and sodium thiosulfate were used in an environmentally friendly manner to investigate the capability of these agents for the leaching of base and precious metals in a single-stage process. Then, a sequential process was employed to leach base metals such as Cu, Pb, and Sn using low concentrated HNO_3_ and HCl and precious metals such as Au, Ag, and Pd using thiourea and sodium thiosulfate from waste motherboards’ PCBs in two consecutive stages.

Screening designs are generally used to identify the most significant parameters from many suspected factors. As far as the authors know, no studies have been carried out on the application of screening designs for the selective leaching of valuable metals from waste PCBs. Therefore, this study is aimed at on one hand comparing the leaching of Cu, Pb and Sn as base metals and Au, Ag and Pd as precious metals from waste motherboards’ PCBs in one and two-stage processes and also to analyze the influence of the variables involved in each stage, namely, leaching agent type and concentration, temperature, solid/liquid ratio, initial pH, average particle size, and time. Mathematical models were developed to describe the impact of the variables mentioned above.

## Materials and methods

### Materials

Waste motherboards’ PCBs were supplied by Revertia (O Porriño, Spain), a company dedicated to the management of waste electrical and electronic equipment. The reagents, sodium hydroxide (NaOH 98%, Sigma Aldrich), hydrochloric acid (HCl 35%, Prolabo), nitric acid (HNO_3_ 65%, Panreac), thiourea (CH_4_N_2_S 99%, Thermo Scientific), and sodium thiosulfate five hydrate (Na_2_S_2_O_3_·5H_2_O 98%, Probus) were used in the present work, which were all of analytical grade.

### Pre-treatment process

Waste PCBs were separated manually from electronic and non-electronic components such as capacitors, resistors, transistors, integrated circuits (ICs), semiconductor chips, slots, and connectors. Then, after washing the bare waste motherboards’ PCBs three times with double distilled water to remove any dirt and other contaminants and drying them in an oven at 80 °C, they were cut manually into small pieces of roughly 2–3 cm in size. Afterwards, the epoxy coating covering PCBs on both sides which is toxic and, moreover, can hinder the contact between the leaching agent (lixiviant) and the metals was eliminated.

Coating removal was performed by a modification of the method used in a preliminary work (Rahimi et al. 2022), applying a 2% aqueous NaOH solution as the leaching agent at a solid/liquid ratio of 200 g L^−1^ under autoclaving treatment (121 °C and 1.1 bar).

After removing the coating, the waste motherboards’ PCBs were crushed using a blade mill (IKA MultiDrive basic, USA) at a rate of 10,000 rpm. Then, the particles obtained were sieved to different particle size ranges (Fig. S1) using a digital electromagnetic sieve shaker (IRIS, Spain) under vibration for 15 min to use them for the leaching experiments and analyze its influence on the leaching process. The reduction in the particle size of PCBs can lead to an increase in the available surface of the solid to be in contact with the leaching agents (Li et al. 2018).

To analyze the initial metal composition (wt%) of PCBs, the particles were treated using three strong acids in three consecutive stages: Stage 1—aqua regia (3 parts of 35% HCl and 1 part of 65% HNO_3_) for 5 h at 80 °C, Stage 2—35% HCl for 3 h at 80 °C, and Stage 3—65% HNO_3_ for 3 h at 80 °C. The stirring rate and the solid/liquid ratio were maintained constant for all stages, 500 rpm and 1/20 (g/mL), respectively. Finally, after determining the concentration of metals in solutions by inductively coupled plasma mass spectrometry (ICP-MS) (Agilent 7700, United States), the initial amount of metals present in PCBs (wt%) was calculated using Eq. (1).

1 $${\text{wt}}\%=\left[\left(\frac{{C}_{i,AR}}{{m}_{PCB,AR}}+\frac{{C}_{i,HCl}}{{m}_{PCB,HCl}}+\frac{{C}_{{i,HNO}_{3}}}{{m}_{{PCB,HNO}_{3}}}\right)\times {V}_{sol}\right]\times {10}^{-4}$$

where ${C}_{i,AR}$, ${C}_{i,HCl}$, and ${C}_{{i,HNO}_{3}}$ (µg L^−1^) are the concentration of metal *i* in aqua regia, hydrochloric acid and nitric acid solutions, respectively, ${m}_{PCB,AR}$, ${m}_{PCB,HCl}$, and ${m}_{{PCB,HNO}_{3}}$ (g) represent the mass of PCBs subjected to leaching using aqua regia, hydrochloric acid, and nitric acid, respectively, and ${V}_{sol}$ (L) refers to the volume of the solutions.

The PCB particles were characterized by different analytical techniques such as SEM, EDX, and XRD analysis. The surface morphology of the particles was obtained using scanning electron microscopy (SEM) (EVO LS15 Zeiss, Germany). In addition, energy-dispersive X-ray spectroscopy (EDX) was used to determine the surface chemical composition of the PCB particles. Moreover, their crystalline structure was analyzed using a micro-X-ray diffractometer (µ-XRD) (Bruker D8 VENTURE PHOTON-III, Germany) in a Kappa geometry, equipped with a sealed Incoatec Iµ S 3.0 microfocus tube (Cu Ka, *λ* = 1.54178 Å) and a multilayer mirror monochromator. The diffractograms were obtained in the angular range of 3–63°, and the data were processed using the Bruker SAINT software package.

### Leaching experiments

#### Screening design for the single-stage recovery of metals

Thiourea and sodium thiosulfate five hydrate were used as leaching agents for the leaching experiments to recover base and precious metals from waste motherboards’ PCBs in an environmentally friendly manner. Leaching experiments were performed by adding the corresponding amount of PCBs to 25 mL of the leaching solutions, which were kept in a water bath orbital shaker (J.P. Selecta Unitronic OR, Spain) at a rate of 100 rpm. The experiments were designed to investigate the impact of six independent variables: leaching agent concentration (*X*_1_, 10–50 g L^−1^), temperature (*X*_2_, 20–60 °C), solid/liquid ratio (*X*_*3*_, 50–150 g L^−1^), initial pH (*X*_*4*_, 8–12 for sodium thiosulfate and 1–5 for thiourea which are stable in basic and acidic environments, respectively (Akcil et al. 2015)), average particle size (*X*_5_, 0.3–1.5 mm) and leaching time (*X*_6_, 4–10 h) on *Y*_1_ (Cu leaching, µg g^−1^), *Y*_2_ (Pb leaching, µg g^−1^), *Y*_3_ (Sn leaching, µg g^−1^), *Y*_4_ (Au leaching, µg g^−1^), *Y*_5_ (Ag leaching, µg g^−1^), and *Y*_6_ (Pd leaching, µg g^−1^), in order to analyze the applicability and ability of these agents in a single-stage leaching process.

Plackett–Burman design as a powerful statistical tool was applied to screen the effect of the independent variables on the leaching of base and precious metals. This design is able to evaluate N-1 independent variables using *N* experiments (*N* must be defined as a multiple of 4) (Mousavi et al. 2018; El-Shafie et al. 2022). The design matrix for a six-variable 12-run Plackett–Burman screening design is shown in Table 1. The experiments were performed at different combinations of high (+ 1) and low (− 1) levels of the process variables to analyze their influence on the leaching process assuming that there is no interaction between parameters and is based on the following first-order model (Eq. (2)):

2 $${Y}_{i}= {A}_{0}+\sum {A}_{i}{X}_{i}$$

where *Y*_*i*_ are the response variables, *A*_0_ is the scaling constant, *A*_*i*_ are the linear regression coefficients of the response variables, and *X*_*i*_ are the independent variables.

**Table 1:** Design matrix generated using Plackett–Burman screening design for single-stage leaching with thiourea and sodium thiosulfate

Run	*X*_1_	*X*_2_	*X*_3_	*X*_4_	*X*_5_	*X*_6_
1	10	20	150	5^a^/12^b^	1.5	4
2	10	60	150	1/8	1.5	4
3	10	20	50	5/12	1.5	10
4	50	20	150	5/12	0.3	10
5	50	20	50	1/8	1.5	10
6	10	60	150	5/12	0.3	10
7	10	20	50	1/8	0.3	4
8	10	60	50	1/8	0.3	10
9	50	60	50	5/12	0.3	4
10	50	60	50	5/12	1.5	4
11	50	60	150	1/8	1.5	10
12	50	20	150	1/8	0.3	4

*X*_1_, leaching agent concentration, g L^−1^; *X*_2_, temperature, °C; *X*_*3*_, solid/liquid ratio, g L^−1^; *X*_*4*_, a: initial pH for thiourea, b: initial pH for sodium thiosulfate; *X*_5_, average particle size, mm; *X*_6_, leaching time, h

#### Screening design for the sequential recovery of base and precious metals

A two-stage process was used for the sequential leaching of base and precious metals. In the first stage, nitric acid and hydrochloric acid were compared to leach base metals such as Cu, Pb and Sn from waste PCBs analyzing the effect of five independent variables: acid concentration (*X*_1_, 2–4 M), temperature (*X*_2_, 30–50 °C), solid/liquid ratio (*X*_*3*_, 30–90 g L^−1^), average particle size (*X*_5_, 0.3–1.5 mm), and leaching time (*X*_6_, 2–6 h) on the amount of Cu, Pb, and Sn leached (*Y*_1_, *Y*_2_, and *Y*_3_, respectively, µg g^−1^). Then, thiourea and sodium thiosulfate five hydrate were used for the second stage to recover precious metals such as Au, Ag, and Pd from the residual solids obtained on a large scale after the first stage at the optimal conditions selected. Previously, the residual materials rich in precious metals were washed by double distilled water to remove any acid remained on the solid surface and then dried at 50 °C for 24 h. The influence of five independent factors: leaching agent concentration (*X*_1_, 10–50 g L^−1^), temperature (*X*_2_, 30–50 °C), solid/liquid ratio (*X*_*3*_, 30–90 g L^−1^), initial pH (*X*_*4*_, 8–10 for sodium thiosulfate and 1–3 for thiourea) and leaching time (*X*_6_, 2–6 h) on *Y*_4_ (Au leaching, µg g^−1^), *Y*_5_ (Ag leaching, µg g^−1^), and *Y*_6_ (Pd leaching, µg g^−1^) was investigated. The optimum average particle size (1.5 mm) determined from the first stage was used for the leaching experiments in the second stage. In order to conduct leaching experiments in both stages, the appropriate quantities of PCBs were added to 50 mL of leaching solutions, which were then shaken at a rate of 250 rpm in an orbital water bath shaker (Hydro H 20, Germany).

To adjust the pH values, HCl and NaOH aqueous solutions (0.1-1 M) were used. Double distilled water was used to prepare all the solutions. After finishing the experiments, the leach liquor solutions were taken, filtered and their metal compositions analyzed using ICP-MS.

For each metal, the amount of metal recovered per unit mass of PCBs (µg g^−1^) (dependent variable) and the leaching efficiency (LE, %) were calculated using Eqs. (3) and (4), respectively.

3 $${Y}_{i,exp}= \frac{{C}_{i}}{{m}_{PCB,in}} \times {V}_{sol}$$

4 $${LE}_{i} \%= \frac{{m}_{i,sol}}{{m}_{i,PCB}} \times 100$$

where *C*_*i*_ (µg L^−1^) is the concentration of metal i in solutions, *m*_*PCB,in*_ (g) is the initial mass of PCBs subjected to leaching, *V*_*sol*_ (L) refers to the volume of the solution, *m*_*i,sol*_ (g) is the mass of metal *i* in the leachate, and *m*_*i,PCB*_ (g) represents the mass of metal *i* in PCBs before leaching.

In the first stage, as mentioned above, five independent variables were screened using the Definitive Screening Design to investigate their impact on the leaching of base metals. The Definitive Screening Design allows to analyze *N* factors by 2*N* + 1 (if *N* is even and greater or equal to 4) and 2*N* + 3 (if *N* is odd and greater than 4) number of experiments (Takagaki et al. 2019). The experiments were conducted at various combinations of low (− 1), medium (0), and high (+ 1) values of the process factors (16 experimental runs) as shown in Table 2 considering the main and quadratic effects for the screening of parameters and is based on the following second-order model (Eq. (5)):

5 $${Y}_{i}= {A}_{0}+\sum {A}_{i}{X}_{i}+\sum {A}_{ii}{X}_{i}^{2}$$

where *A*_*ii*_ are the quadratic regression coefficients. All the experiments were performed in duplicate, and the average value of the dependent variables was used for data analysis.

**Table 2:** Design matrix generated using Definitive Screening Design for the acid leaching stage using HNO_3_ and HCl

Run	*X*_1_	*X*_2_	*X*_*3*_	*X*_5_	*X*_6_
1	2	30	90	0.9	2
2	3	40	60	0.9	4
3	3	30	30	0.3	2
4	3	50	90	1.5	6
5	4	50	30	0.9	6
6	2	40	30	0.3	6
7	3	40	60	0.9	4
8	4	40	90	1.5	2
9	3	40	60	0.9	4
10	2	50	90	0.3	4
11	4	30	30	1.5	4
12	4	30	90	0.3	6
13	4	50	60	0.3	2
14	3	40	60	0.9	4
15	2	30	60	1.5	6
16	2	50	30	1.5	2

*X*_1_, acid concentration, M; *X*_2_, temperature, °C; *X*_*3*_, solid/liquid ratio, g L^−1^;* X*_5_, average particle size, mm; *X*_6_, leaching time, h

Definitive Screening Design can be also used for optimization of response variables considering the interactions between independent variables with a significantly low number of experiments. Therefore, the interaction term ($\sum {A}_{jk}{X}_{j}{X}_{k}$) can be added to Eq. (5) to construct a complete second order model (Eq. (6)) for optimization using the three most important factors affecting the leaching process selected from the previous analysis.

6 $${Y}_{i}= {A}_{0}+\sum {A}_{i}{X}_{i}+\sum {A}_{ii}{X}_{i}^{2}+\sum {A}_{jk}{X}_{j}{X}_{k}$$

where *A*_*jk*_ are the regression coefficients for the double interactions.

For the second stage, a quarter fraction screening design with a lower number of experiments compared to the other two designs (Plackett–Burman and Definitive Screening Designs) was used to screen the influence of the five independent variables previously mentioned on the recovery of precious metals based on a linear model similar to the one presented in Eq. (2). Five factors were screened by performing 8 experiments (2^(*N*−2)^ experimental runs) at different combinations of high and low levels as can be seen in Table 3. All the experiments were carried out in triplicate and the mean value of the dependent variables was used to analyze the data.

**Table 3:** Design matrix for the quarter fraction screening design for the second leaching stage using thiourea and sodium thiosulfate

Run	*X*_1_	*X*_2_	*X*_*3*_	*X*_*4*_	*X*_6_
1	10	50	30	1^a^/8^b^	6
2	10	30	90	3/10	2
3	10	30	30	3/10	6
4	50	30	90	1/8	6
5	10	50	90	1/8	2
6	50	30	30	1/8	2
7	50	50	90	3/10	6
8	50	50	30	3/10	2

*X*_1_, leaching agent concentration, g L^−1^; *X*_2_, temperature, °C; *X*_*3*_, solid/liquid ratio, g L^−1^; *X*_*4*_, a: initial pH for thiourea, b: initial pH for sodium thiosulfate; *X*_6_, leaching time, h

### Data analysis

The design of experiments and the analysis of the experimental results were conducted using Statgraphics Version 18. Evaluation of the significant variables affecting on the responses was carried out at the 95.0% confidence level using *p*-values from the regression analysis. The independent variables with a *p*-value lower than 0.05 were found to be statistically significant possessing substantial impacts on the response variables. Moreover, the significancy of the models obtained was analyzed based on a *p*-value less than 0.05 and high coefficient of determination (*R*^2^).

## Results and discussion

### Raw material preparation and characterization

As mentioned before, after removing the electronic and non-electronic components present on the PCBs and cutting them into small pieces, an alkaline pre-treatment was carried out using a 2% aqueous NaOH solution to eliminate the epoxy coating present on the surface of the PCBs considering that very small amounts of valuable metals were dissolved in the NaOH solution. Thus, a significant amount of Al (5205 mg kg^−1^) was leached in the NaOH solution, whereas only 515 and 24 mg kg^−1^ of Sn and Cu were dissolved, respectively, which were low compared to the initial amount of the metals present in PCBs. The lixiviated amounts of other metals were lower than 5 mg kg^−1^ (3.95, 1.06, 0.26, 0.25, 0.01, 0.005, and 0.49 mg kg^−1^ for Ni, Zn, Pb, Sb, Pd, Ag, and Au, respectively).

Subsequently, the PCBs grinding was performed to reduce their size and improve the contact between the metals and the leaching agents. The particles with a size of 0.1–0.5 mm had the highest weight percentage (31.41%) followed by 1–2 mm (25.14%), 0.5–1 mm (24.56%), and < 0.1 mm (18.89%). The particle sizes of 0.1–0.5, 0.5–1, and 1–2 mm were used for the leaching experiments.

Once PCBs were free of coating, ground and separated by particle size, they were treated with three concentrated acids (aqua regia, HCl, and HNO_3_) in three sequential stages, as indicated previously, in order to analyze and estimate the initial content of metals present in each PCB particle size as can be seen in Table 4. As can be observed, copper is the main component with significant quantities followed by tin and different amounts of other metals.

**Table 4:** Initial metal composition of waste motherboards’ PCBs (wt %)

	Metals															
Particle size, mm	Na	Mg	Al	K	Ca	Fe	Ni	Cu	Zn	Sn	Pb	Sb	Pd	Ag	Au	R
0.1–0.5	1.409	0.188	1.723	0.053	3.713	0.091	0.085	37.942	0.462	4.144	1.163	0.006	0.0005	0.056	0.002	48.96
0.5–1.0	2.095	0.231	1.325	0.069	3.359	0.073	0.295	57.167	5.127	3.885	1.009	0.006	0.0003	0.068	0.003	25.29
1.0–2.0	1.661	0.208	1.698	0.056	4.299	1.002	0.101	54.652	1.167	6.247	0.296	0.009	0.0002	0.058	0.001	28.54

R is residue corresponding to non-metals (wt%)

It is important to highlight that these values are considered an estimate since it is difficult to accurately determine the metal content in the ground PCB samples due to their heterogeneity, so that its determination is highly dependent on the particle size analyzed and the mass of sample used (Touze et al. 2020).

**Fig. 1:**
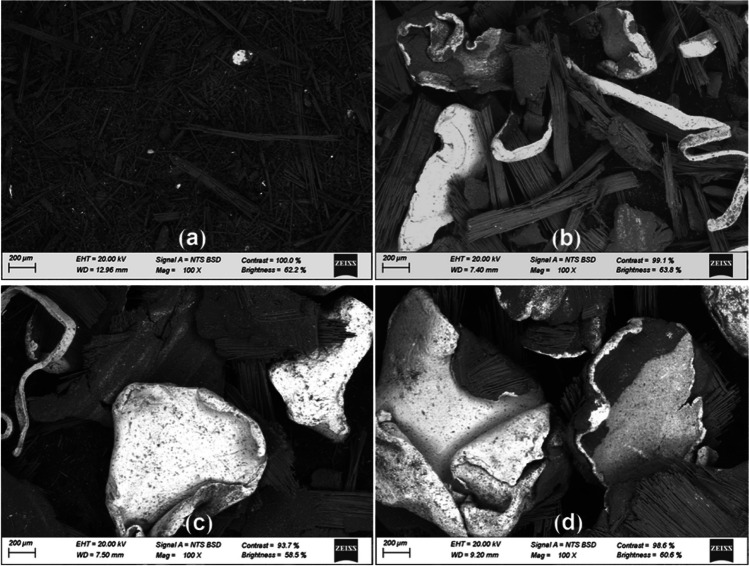
SEM images corresponding to different PCB particle size ranges: **a** < 0.1 mm, **b** 0.1–0.5 mm, **c** 0.5–1.0 mm, and **d** 1.0–2.0 mm

The surface morphological analysis (SEM) was conducted for the different sizes of PCB particles (Fig. 1). As observed, the PCB particles exhibit irregular shapes that confirm a high degree of heterogeneity that increases with increasing the particle size. In addition, an agglomeration of particles is observed for those of size lower than 0.1 mm which can hinder the contact between metals and leaching agents and consequently lead to a reduction in metal recovery (Wu et al. 2008). The different colors, white and grey, shown in Fig. 1 are related to the presence of metallic and non-metallic solid fractions, respectively.

The findings for the surface chemical composition of PCBs from EDX analysis are presented in Table 5. These data were obtained from EDX spectra for some specific areas of PCB surfaces as shown in Figs. S2-S5, and an average value was calculated for each particle size. As can be observed, the particles with size lower than 0.1 mm showed a significantly lower quantity of Cu (1.7%) than the other size ranges (21.3, 18.6, and 13.1% for 0.1–0.5, 0.5–1.0, and 1.0–2.0 mm, respectively). No precious metals (Au, Ag, and Pd) were found in the surfaces analyzed probably due to the heterogeneity of particles and the low amount of these metals in comparison with copper, the most abundant, and other metals such as Sn and Pb.

**Table 5:** Surface chemical composition of PCB particles obtained using EDX analysis (wt %) for the different PCB particle sizes

	Components														
Particle size, mm	C	O	Al	Si	Ca	Ti	Cr	Fe	Ni	Cu	Zn	Br	Sn	Ba	Pb
< 0.1	44.6	27.0	1.8	8.7	7.4	0.2	0.1	0.4	0.1	1.7	–	7.3	0.5	–	–
0.1–0.5	45.1	15.1	0.9	4.5	2.8	–	0.1	0.2	0.1	21.3	0.1	7.7	1.5	–	0.5
0.5–1.0	47.5	14.1	1.2	3.6	2.2	–	–	0.20	0.6	18.6	0.7	7.6	2.3	0.1	1.1
1.0–2.0	54.9	15.7	0.5	3.2	2.0	–	0.1	0.2	–	13.1	0.3	6.4	2.2	–	1.3

**Fig. 2:**
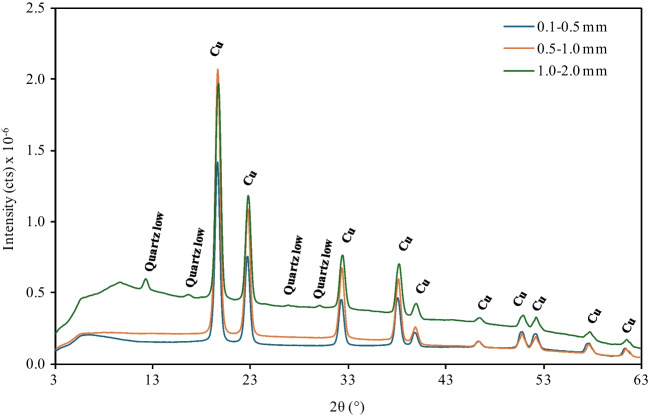
µ-XRD diffractograms of PCB particles for the different size ranges employed

X-ray diffraction analysis was carried out to structurally characterize the PCB particles. Due to the significant heterogeneity of the samples, their composition was individually determined at microscopic level and the results obtained are illustrated in Fig. 2. It is observed that the µ-XRD patterns are very similar exhibiting the same peaks for all particle sizes. Most of the peaks correspond to copper, since this metal is the most abundant in waste PCBs which are known as copper-based materials (Hossain et al. 2019). Moreover, some weak peaks corresponding to the presence of quartz low (SiO_2_) were only observed for the larger particles. The metallic and fibrous samples analyzed showed a transparent-white appearance indicating very weak diffraction, typical of poorly crystalline systems (Fig. S6) (Ichikawa et al. 2023).

### Single-stage leaching experiments

A Plackett–Burman screening design was used for planning the leaching experiments to compare the recovery of base (Cu, Pb and Sn) and precious metals (Au, Ag and Pd) in a single-stage process using thiourea or sodium thiosulfate as leaching agents considering six independent variables affecting the process (Table 1). The results obtained for the recovery of base and precious metals leached from waste motherboards’ PCBs (in µg of metal/g of PCBs) (Table 6) showed that low amounts of all the metals were leached using both agents, especially for precious metals. For instance, the highest leaching efficiencies for Cu, Pb, and Sn were 19.6% (106,962 µg g^−1^), 17.5% (2039.5 µg g^−1^), and 2.8% (1164.7 µg g^−1^), respectively, and were obtained using thiourea. In the case of precious metals, the maximum Au, Ag, and Pd leaching efficiencies were also achieved using thiourea and were 0.64% (0.0988 µg g^−1^), 0.44% (2.55 µg g^−1^), and 9.9% (0.195 µg g^−1^), respectively. In general, thiourea seems to be a more efficient leaching agent compared to sodium thiosulfate to recover base and precious metals in the conditions essayed.

**Table 6:** Experimental values for the amount of metal recovered per unit mass of PCBs using thiourea and sodium thiosulfate in a single-stage process

	Thiourea						Sodium Thiosulfate					
Run	*Y*_1_ (µg g^−1^)	*Y*_2_ (µg g^−1^)	*Y*_3_ (µg g^−1^)	*Y*_4_ (µg g^−1^)	*Y*_5_ (µg g^−1^)	*Y*_6_ (µg g^−1^)	*Y*_1_ (µg g^−1^)	*Y*_2_ (µg g^−1^)	*Y*_3_ (µg g^−1^)	*Y*_4_ (µg g^−1^)	*Y*_5_ (µg g^−1^)	*Y*_6_ (µg g^−1^)
1	676.963	0.0095	0.4362	0.0000	0.0000	0.0000	211.771	0.0044	0.3345	0.0172	0.1794	0.1201
2	12,638.7	590.22	69.904	0.0000	0.2576	0.0139	4583.68	0.0470	0.0385	0.0000	0.0000	0.0011
3	1428.87	0.0259	0.3839	0.0000	0.0125	0.0000	269.011	0.0000	0.1884	0.0676	0.8432	0.1074
4	2830.10	0.0106	0.6979	0.0000	0.1052	0.0015	3749.13	0.2800	1.8467	0.0204	0.5521	0.0490
5	106,962	66.949	0.7661	0.0000	1.4600	0.1951	14,192.3	0.0000	2.3931	0.0726	0.2590	0.0315
6	1561.53	0.3020	0.0222	0.0000	0.0025	0.0006	66.4960	11.838	0.5739	0.0000	0.0000	0.0019
7	22,391.5	2039.5	1164.7	0.0988	1.0054	0.1690	2551.85	0.0000	0.0919	0.0820	0.4654	0.0303
8	28,506.7	1175.0	1045.2	0.0000	0.9872	0.0726	20,543.4	0.4029	0.0430	0.0000	0.0000	0.0035
9	14,055.4	0.0827	0.2884	0.0000	0.6632	0.0000	11,382.0	1.7183	26.675	0.0048	2.4194	0.0665
10	13,057.6	0.2659	0.1359	0.0000	0.0664	0.0000	11,816.3	1.9720	76.004	0.0070	1.0402	0.0324
11	63,996.1	70.689	3.5361	0.0000	2.5523	0.0487	7117.25	0.0749	23.773	0.0035	0.7803	0.0193
12	20,934.6	7.1865	12.034	0.0000	0.0689	0.0121	4062.39	0.2578	2.4497	0.0395	0.3879	0.0278

*Y*_1_, *Y*_2_, *Y*_3_, *Y*_4_, *Y*_5_, and *Y*_6_ correspond to Cu, Pb, Sn, Au, Ag, and Pd recovery, respectively

The regression analysis of the models obtained by Plackett–Burman design for the recovery base and precious metals using thiourea and sodium thiosulfate as leaching agents is shown in Tables S1 and S2. As seen, initial pH is the only significant parameter for the recovery of Cu, Pb, and Pd using thiourea having a negative influence. For sodium thiosulfate, concentration with a positive impact, temperature, and solid/liquid ratio with negative effects and initial pH having a positive influence were found to be significant for the recovery of Sn, Au, and Pd, respectively.

The low recovery of precious metals using these leaching agents in a single-stage process could be related to the presence of large amounts of base metals, particularly Cu in PCBs, which can prevent the contact between the leaching agents and precious metals. However, it has been proven that thiourea and sodium thiosulfate are suitable leaching agents for the leaching of precious metals such as Au and Ag (Jing-ying et al. 2012; Alvarado-Macias et al. 2016). Therefore, the decision was to leach base and precious metals in two consecutive stages.

### Two-stage sequential leaching experiments

A two-stage sequential leaching process was then proposed to recover base (Cu, Pb, and Sn) and precious metals (Au, Ag, and Pd) from waste motherboards’ PCBs. Experimental designs were used to screen the effect of parameters on the leaching of metals in both stages and to optimize the first stage.

#### First stage—leaching of base metals

Nitric acid (HNO_3_) and hydrochloric acid (HCl) were used in the first stage focused on the leaching of Cu, Pb, and Sn as base metals from PCBs. After performing the leaching experiments designed using a Definitive Screening Design (Table 2), in general, HNO_3_ was found to be much more efficient than HCl for the recovery of Cu and Pb under the conditions studied as shown in Table 7, whereas a considerably higher amount of Sn was leached using HCl reaching a maximum leached amount of 71,584.5 µg g^−1^ (leaching efficiency > 100% using the estimated composition included in Table 4) corresponding to experiment 11 (being 8380.9 µg g^−1^ (5.7%) using HNO_3_ in experiment 3). Yoo et al. (2012) also reported extremely low amounts of Sn leached using HNO_3_ as leaching agent.

**Table 7:** Experimental values for the amount of base and precious metals recovered per unit mass of PCBs using nitric acid and hydrochloric acid

	HNO_3_						HCl					
Run	*Y*_1_ (µg g^−1^)	*Y*_2_ (µg g^−1^)	*Y*_3_ (µg g^−1^)	*Y*_4_ (µg g^−1^)	*Y*_5_ (µg g^−1^)	*Y*_6_ (µg g^−1^)	*Y*_1_ (µg g^−1^)	*Y*_2_ (µg g^−1^)	*Y*_3_ (µg g^−1^)	*Y*_4_ (µg g^−1^)	*Y*_5_ (µg g^−1^)	*Y*_6_ (µg g^−1^)
1	155,971.7	15,004.8	507.5	1.49	0.24	0.08	15.9	4609.8	20,462.0	0.01	7.11	0.02
2	362,195.2	13,229.2	848.1	4.60	3.45	1.36	23.7	11,732.1	64,667.9	0.16	14.14	0.22
3	225,995.3	16,220.8	8380.9	1.06	1.32	0.40	2000.9	10,082.7	42,911.6	0.17	98.24	0.13
4	355,418.4	22,932.5	348.1	9.76	0.74	1.08	0.5	8317.5	43,593.8	0.00	2.74	0.07
5	406,062.6	25,021.4	2024.2	10.75	1.66	1.57	79,149.8	8559.9	57,246.7	0.53	91.39	0.75
6	197,323.9	12,607.9	1894.4	2.97	2.83	1.69	108,864.8	10,966.5	42,944.9	0.09	83.25	0.19
7	389,850.6	15,911.3	236.9	6.56	1.16	1.32	0.0	10,708.2	56,054.5	0.00	9.87	0.06
8	342,177.5	26,057.6	4438.2	1.72	0.70	0.90	125.5	5044.4	26,717.8	0.04	12.89	0.07
9	422,504.6	20,491.5	633.8	7.50	2.24	1.05	405.3	11,348.8	56,399.7	0.00	9.13	0.05
10	213,280.0	13,614.7	76.4	4.15	0.19	3.00	198.2	7310.0	31,271.0	0.00	11.54	0.10
11	360,903.4	19,630.0	3587.3	1.40	0.99	0.80	6897.8	3693.1	71,584.5	0.11	30.79	0.12
12	247,219.1	16,804.1	5691.3	4.13	0.28	1.51	2054.6	10,864.5	43,866.0	0.01	32.02	0.07
13	244,578.3	15,654.9	3305.2	2.57	0.68	2.14	2495.0	9291.0	38,803.1	0.01	36.00	0.12
14	390,480.4	18,003.3	1762.1	8.41	1.81	1.53	0.0	10,072.5	46,398.6	0.00	7.31	0.05
15	317,172.3	11,453.7	467.4	2.42	2.45	0.87	458.8	5540.7	45,647.0	0.01	7.52	0.04
16	301,937.3	32,109.4	160.6	6.50	0.88	1.22	8260.4	3084.1	39,697.0	0.00	20.57	0.08

*Y*_1_, *Y*_2_, *Y*_3_, *Y*_4_, *Y*_5_, and *Y*_6_ correspond to Cu, Pb, Sn, Au, Ag, and Pd recovery, respectively

For Cu which is the base metal with the greatest concentration in PCB waste, the highest leached amount was 422,504.6 µg g^−1^ (73.9%) using HNO_3_ (experiment 9) compared to 108,864.8 µg g^−1^ (28.7%) using HCl (experiment 6) as seen in Table 7. Moreover, extremely low or no presence of Cu was observed in some experiments with HCl as has been reported in other studies (Moosakazemi et al. 2019; Sapinov et al. 2020). These results provide important information in relation to the use of HCl or HNO_3_ in a selective way depending on the target metals.

Table 7 shows that the amounts of Pb leached using HNO_3_ (all the experiments) and HCl (some experiments) as well as the Sn leached in some experiments using HCl are higher than those obtained in the initial estimated composition of the PCBs (Table 4), which could be explained by the heterogeneity of PCB particles in relation to these metals since as they are used for soldering electronic parts on the PCBs surface, some pieces may remain with concentrated amounts of these metals and affect sample homogeneity (Li et al. 2018; Touze et al. 2020). As indicated above, the highest leached quantity of Pb was achieved using HNO_3_, 32,109.4 µg g^−1^ (experiment 16), in comparison with 11,732.1 µg g^−1^ (experiment 2) for HCl. In particular, the removal of this metal from waste PCBs possesses prominent benefits as it is considered a threat to both human health and the environment.

In the case of precious metals, whose leached quantity should be minimized in this stage in order to extract them in the second stage, in general, the recovery of Au and Pd was higher using HNO_3_ than using HCl, which was a more suitable lixiviant for the recovery of Ag (Table 7). Despite the highest amounts of Au and Pd leached using HNO_3_ were 10.75 (38.3%) and 3.00 µg g^−1^ (64.2%), respectively, it is important to highlight that the values for the amount of these metals in the leachate present a high variability depending on the conditions used, therefore, to achieve the minimum amount, the conditions in which the leaching process is carried out must be adjusted and controlled. Based on the results obtained, HNO_3_ was selected as a more appropriate agent for the leaching of base metals, especially Cu and Pb in the first stage due to their environmental and economical importance.

Hence, the influence of the five independent variables selected (HNO_3_ concentration, temperature, solid/liquid ratio, average particle size, and leaching time) on the amount of metal recovered per unit mass of PCBs using HNO_3_ as leaching agent (*Y*_1_, *Y*_2_, *Y*_4_, *Y*_5_, and *Y*_6_, for Cu, Pb, Au, Ag, and Pd, respectively) was analyzed using the Definitive Screening Design planned to determine the significant parameters. The goal was set to maximize the leaching of base metals (Cu and Pb) and minimize the leaching of precious metals (Au, Ag, and Pd) to maintain the material rich in precious metals and use for the second stage.

The regression analysis of the models obtained by the Definitive Screening Design for the recovery of Cu, Pb, Au, Ag, and Pd using nitric acid as the leaching agent is shown in Table 8. As seen, nitric acid concentration and average particle size were found to be significant having positive impacts on the leaching of Cu. This means that increasing the HNO_3_ concentration and average particle size can lead to a rise in the recovery of Cu in the range studied. The high acid strength produced by increasing the HNO_3_ concentration can result in an increase in the leaching rate of Cu (Mecucci and Scott 2002). In addition, leaching time was considered as a significant factor since its *p*-value was close to 0.05 (*p*-value = 0.0701).

**Table 8:** Regression analysis of the models obtained for the recovery of metals using nitric acid

Regression analysis of the independent variables										
	Regression coefficient					*p*-value				
Term	*Y*_1_	*Y*_2_	*Y*_4_	*Y*_5_	*Y*_6_	*Y*_1_	*Y*_2_	*Y*_4_	*Y*_5_	*Y*_6_
Constant	− 732,564	− 710.277	− 14.2429	− 12.6815	0.260613					
*X*_1_—HNO_3_ concentration (M)	250,105	8329.47	13.2508	0.494087	− 3.02045	0.0130	0.2378	0.6090	0.3970	0.9375
*X*_2_—temperature (°C)	13,185.8	972.585	− 0.491294	0.673508	0.090182	0.1093	0.0787	0.0089	0.6685	0.0006
*X*_3_—solid/liquid ratio (g L^−1^)	2730.37	− 695.977	− 0.105482	0.069192	0.037436	0.1662	0.4520	0.8111	0.0752	0.2579
*X*_5_—average particle size (mm)	216,903	13,805.2	15.5071	0.27442	− 2.74617	0.0041	0.0418	0.2702	0.8597	0.0025
*X*_6_—leaching time (h)	74,923	− 4556.13	− 0.105174	− 0.430334	1.40272	0.0701	0.2898	0.0307	0.1542	0.0348
*X*_1_^2^	− 34,763.2	− 1081.95	− 2.15767	− 0.120361	0.504355	0.1831	0.7156	0.1179	0.8207	0.0159
*X*_2_^2^	− 138.071	− 8.37988	0.009044	− 0.008559	− 0.000458	0.5664	0.7772	0.4649	0.1502	0.7584
*X*_*3*_^2^	− 27.7019	5.48937	0.000840	− 0.00073	− 0.000288	0.3184	0.1385	0.5379	0.2492	0.1259
*X*_5_^2^	− 69,648.8	− 4217.65	− 7.97353	− 0.110024	1.16722	0.3159	0.6115	0.0538	0.9404	0.0308
*X*_6_^2^	− 7787.03	468.093	0.117399	0.079621	− 0.162958	0.2249	0.5340	0.6984	0.5552	0.0057
Regression analysis of the response variables										
Determination coefficient (*R*^2^)	0.9420	0.8046	0.8937	0.7868	0.9657					
*p*-value	0.0161	0.2201	0.0632	0.2589	0.0046					

*Y*_1_, *Y*_2_, *Y*_4_, *Y*_5_, and *Y*_6_ (µg g^−1^) correspond to Cu, Pb, Au, Ag, and Pd recovery, respectively. Significant effects and models are underlined

In the case of Pb, only average particle size with a positive effect was found to be significant, but temperature was also considered significant due to the proximity of its *p*-value to 0.05 (*p*-value = 0.0787) having a positive impact on the metal leaching.

In relation to the leaching of Au, it was observed that temperature and leaching time are significant parameters on the response both possessing negative influence. This means that increasing temperature and leaching time the recovery of Au increases. Moreover, since the *p*-value of the quadratic term of average particle size is very close to 0.05 (*p*-value = 0.0538), this effect was also considered significant with a negative impact on the response.

No variable had a *p*-value lower than 0.05 for the leaching of Ag, but solid/liquid ratio with a positive effect was considered a significant parameter due to the closeness of its *p*-value to 0.05 (*p*-value = 0.0752).

Regarding Pd leaching, two variables, temperature and leaching time, with positive impacts and average particle size with a negative effect are significant. Furthermore, the quadratic terms of HNO_3_ concentration, average particle size, and leaching time were found to be significant.

In general, average particle size was found to be the most important parameter influencing the Cu, Pb, and Pd leaching. As particle size increases, it seems that greater contact between metals and the leaching solution is facilitated resulting in an increase of recovery of desired metals. However, since the target in this stage was to minimize the leaching of Pd, the lower particle size must be selected.

As mentioned above, temperature and leaching time were found to be significant for the recovery of precious metals (Au and Pd). With the rise in temperature and leaching time, the interaction between Au and HNO_3_ increases leading to an acceleration in the diffusion rate and then an increase in the Au leaching (Javed et al. 2018). However, this behavior is opposite for Pd. The very low amount of Pd present in waste PCBs and the increased interaction of metals can promote the leaching of other active metals such as Cu and Au that compete with Pd, and Pd recovery decreases.

The probability (*p*-values) and the determination coefficient (*R*^2^) for the responses (Table 8) demonstrated that only models for Cu and Pd are significant. Moreover, the model obtained for Au recovery was also considered significant for having a *p*-value close to 0.05. Fig. S7 shows the observed versus predicted values by the Definitive Screening Design for the recovery of Cu, Pb, Au, Ag, and Pd confirming the goodness of the models for Cu, Pd, and Au.

With the aim of optimizing metal recovery, the variables with the greatest influence on Cu, Au, and Pd leaching were chosen, and the regression analysis was carried out according to Eq. (6). Although only one and two important variables were found for the recovery of Ag and Pb, respectively, and the models for these responses were insignificant, these metals were also considered for optimization together with the above-mentioned metals since the interaction between these metals (Pb and Ag) and HNO_3_ can affect the leaching of other metals (Cu, Au, and Pd) causing a change in the variables optimized. Hence, in the case of Cu, HNO_3_ concentration, average particle size, and leaching time were selected; for Pb, temperature, average particle size, and solid/liquid ratio (due to the quadratic effect); for Au, temperature, leaching time and average particle size; for Ag, temperature (due to the quadratic effect), solid/liquid ratio and leaching time; and for Pd, temperature, average particle size, and leaching time were selected.

The optimization was carried out for all the response variables together to maximize the leaching of Cu and Pb as base metals and minimize the leaching of Au, Ag and Pd as precious metals considering the interactions and quadratic effects. The dependability of the models was investigated using the regression analysis as shown in Table 9. The models present a *R*^2^ value higher than 0.8 (except for Ag) indicating a high correlation between observed and predicted values. As previously discussed, the models are statistically significant if the *p*-value is lower than 0.05. Hence, the models obtained for the leaching of Cu, Au, and Pd were found to be significant. In the case of Pb and Ag, *p*-values greater than 0.05 indicate insignificancy of these models. The regression models fitted with the experimental data corresponding to the leaching of Cu, Au, and Pd (significant models using the variables with the highest impact) using Definitive Screening Design for optimization are shown in Eqs. (7)–(9), respectively.

7 $${Y}_{1}= -784901+378407{X}_{1}+ 428907{X}_{5}$$

8 $${Y}_{4}= 3.96-0.59{X}_{2}+2.37{X}_{5}+3.38{X}_{6}-8.18{X}_{5}^{2}+0.30{X}_{2}{X}_{5}$$

9 $${Y}_{6}= -3.03+0.03{X}_{2}-0.111{X}_{5}+1.4{X}_{6}-0.107{X}_{6}^{2}$$

where *X*_1_, *X*_2_, *X*_5_, and *X*_6_ are HNO_3_ concentration (M), temperature (°C), average particle size (mm), and leaching time (h), respectively. Regarding the interaction effects of variables on the recovery of Cu, Au, and Pd, only the interaction between temperature and average particle size (*X*_2_*X*_5_) was found to be significant on the Cu recovery (Table 9).

**Table 9:** Regression analysis obtained for the optimization of response variables using Definitive Screening Design

Regression analysis of the independent variables										
	Regression coefficient					*p*-value				
Term	*Y*_1_	*Y*_2_	*Y*_4_	*Y*_5_	*Y*_6_	*Y*_1_	*Y*_2_	*Y*_4_	*Y*_5_	*Y*_6_
Constant	− 784,901	− 1716.67	3.96475	− 10.8716	− 3.03216					
*X*_1_—HNO_3_ concentration (M)	378,407	–	–	–	–	0.0312	–	–	–	–
*X*_2_—temperature (°C)	–	1115.84	− 0.592901	0.618599	0.03045	–	0.0513	0.0011	0.6541	0.0003
*X*_3_—solid/liquid ratio (g L^−1^)	–	− 224.466	–	0.082882	–	–	0.4038	–	0.0592	–
*X*_5_—average particle size (mm)	428,907	− 10,399.3	2.37006	–	− 0.110708	0.0101	0.0242	0.1312	–	0.0014
*X*_6_—leaching time (h)	143,404	–	3.3801	− 0.729594	1.40401	0.1396	–	0.0056	0.1327	0.0291
*X*_1_^2^	− 45,766.4	–	–	–	–	0.2046	–	–	–	–
*X*_2_^2^	–	− 12.1345	0.007811	− 0.008435	0.001112	–	0.6687	0.3986	0.1527	0.4879
*X*_*3*_^2^	–	3.43089		− 0.000921	–	–	0.2961	–	0.1586	–
*X*_5_^2^	− 108,959	1517.3	− 8.18412	–	0.733032	0.2684	0.8463	0.0140	–	0.1302
*X*_6_^2^	− 11,928.4	–	− 0.265752	0.092808	− 0.106643	0.1885	–	0.2624	0.4982	0.0298
*X*_1_*X*_5_	− 31,957.2	–	–	–	–	0.3726	–	–	–	–
*X*_1_*X*_6_	− 8380.28	–	–	–	–	0.4320	–	–	–	–
*X*_2_*X*_3_	–	− 4.09965	–	0.000330	–	–	0.4892	–	0.7669	–
*X*_2_*X*_5_	–	447.886	0.303561	–	− 0.034688	–	0.1587	0.0141		0.0669
*X*_2_*X*_6_	–	–	− 0.018229	0.006308	− 0.008672	–	–	0.5187	0.7060	0.1120
*X*_3_*X*_5_	–	− 67.2307	–	–	–	–	0.4959	–	–	–
*X*_3_*X*_6_	–	–	–	− 0.000976	–	–	–	–	0.8604	–
*X*_5_*X*_6_	− 11,343.3	–	0.343429	–	− 0.116599	0.5196	–	0.4679	–	0.1839
Regression analysis of the response variables										
Determination coefficient (*R*^2^)	0.8734	0.8068	0.9358	0.7619	0.9582					
*p*-value	0.0385	0.1128	0.0060	0.1844	0.0018					

*Y*_1_, *Y*_2_, *Y*_4_, *Y*_5_, and *Y*_6_ (µg g^−1^) correspond to Cu, Pb, Au, Ag, and Pd recovery, respectively. Significant effects and models are underlined

Finally, the optimum setting of leaching variables was found to be HNO_3_ concentration (3.4 M), temperature (35°C), solid/liquid ratio (90 g L^−1^), average particle size (1.5 mm), and leaching time (2 h). These values were obtained with the aim of maximizing the leaching of Cu and Pb and minimizing that of Au, Ag, and Pd as previously mentioned. At these optimal conditions, the amounts of Cu, Pb, Au, Ag, and Pd leached were 355,955 (65%), 19,250 (˃100%), 0, 0.64 (0.11%), and 0.47 (24%) µg g^−1^, respectively. Thus, a high recovery of Cu and Pb together with a significantly low amount of precious metals leached was achieved in the first stage to possess raw material rich in precious metals, which are expected to be recovered in the second leaching stage using thiourea and sodium thiosulfate.

### Second stage—leaching of precious metals

As mentioned above, thiourea and sodium thiosulfate were used as two promising leaching agents with low environmental impacts to leach precious metals (Au, Ag, and Pd) from the solids obtained after nitric acid leaching of base metals in the first stage. The Quarter Fraction screening design was employed to analyze the effect of five selected factors, leaching agent concentration (10–50 g L^−1^), temperature (30–50°C), solid/liquid ratio (30–90 g L^−1^), initial pH (1–3 for thiourea and 8–10 for sodium thiosulfate), and leaching time (2–6 h) on the response variables. The mean of the experimental values for the amount of precious metals recovered per unit mass of PCBs using thiourea and sodium thiosulfate according to the Quarter Fraction Design applied are given in Table 10. It can be observed that thiourea is able to leach a higher amount of Au than sodium thiosulfate reaching a maximum value of 0.7521 µg g^−1^ (6.02%, experiment 5). The maximum value using sodium thiosulfate was 0.1911 µg g^−1^ (1.53%) corresponding to experiment 7. In the case of Ag, approximately the same results were obtained for both leaching agents as the highest amounts of Ag leached using thiourea and sodium thiosulfate were 245.8 (16.94%) and 258.0 µg g^−1^ (17.78%), respectively. In addition, sodium thiosulfate is a better agent than thiourea to recover Pd reaching the maximum leached amount of 0.5841 µg g^−1^ (30.10%, experiment 6) compared to 0.3299 µg g^−1^ (17.00%, experiment 4 for thiourea).

**Table 10:** Experimental values for the amount of precious metals recovered per unit mass of PCBs using thiourea and sodium thiosulfate in the second stage

	Thiourea			Sodium thiosulfate		
Run	*Y*_4_ (µg g^−1^)	*Y*_5_ (µg g^−1^)	*Y*_6_ (µg g^−1^)	*Y*_4_ (µg g^−1^)	*Y*_5_ (µg g^−1^)	*Y*_6_ (µg g^−1^)
1	0.6861	178.7	0.2562	0.1296	207.6	0.4562
2	0.3066	194.2	0.2379	0.1058	224.5	0.4989
3	0.3833	227.7	0.2819	0.1061	224.2	0.4982
4	0.7502	245.8	0.3299	0.1338	214.7	0.5089
5	0.7521	221.3	0.3147	0.1193	220.8	0.4703
6	0.4865	233.5	0.2671	0.1443	258.0	0.5841
7	0.7455	230.7	0.2712	0.1911	238.6	0.5572
8	0.3246	199.7	0.1970	0.1311	220.9	0.5017

*Y*_4_, *Y*_5_, and *Y*_6_ correspond to Au, Ag, and Pd recovery, respectively

In general, thiourea and sodium thiosulfate were found to leach significant amounts of precious metals after acid leaching of base metals in comparison to the amounts leached in a single-stage leaching process using these two leaching agents (Table 6). This is because a preliminary acid treatment of waste motherboards’ PCBs can reduce the agglomeration of base metals (especially Cu) allowing precious metals to contact with these agents.

After obtaining the quantities of precious metals leached, the statistical analysis was carried out using Statgraphics Version 18 in order to determine the significant variables involved in the leaching processes using thiourea and sodium thiosulfate together with their effect for each metal. The results are presented in Tables 11. and 12. for thiourea and sodium thiosulfate, respectively. Initial pH is the only significant parameter on precious metals leaching having a negative impact on the leaching of Au by thiourea. This means that a reduction in initial pH can lead to an increase in the recovery of Au, which is in agreement with the results obtained by Xu et al. (2020). This is because thiourea is able to maintain its stability in very acidic environment and as the initial pH increases, it may be decomposed so that the decomposition can occur instantly in basic environment (Xu et al. 2020).

**Table 11:** Regression analysis of the models obtained for the recovery of precious metals using thiourea in the second stage

Regression analysis of the independent variables						
	Regression coefficient			*p*-value		
Term	*Y*_4_	*Y*_5_	*Y*_6_	*Y*_4_	*Y*_5_	*Y*_6_
Constant	0.116404	220.51	0.289532			
*X*_1_—leaching agent concentration (g L^−1^)	0.001117	0.549247	− 0.000159	0.4886	0.3787	0.8278
*X*_2_—temperature (°C)	0.007272	− 0.885019	− 0.000970	0.1114	0.4617	0.5304
*X*_3_—solid/liquid ratio (g L^−1^)	0.002808	0.218409	0.000631	0.0865	0.5725	0.2796
*X*_4_—initial pH	− 0.11438	− 3.40188	− 0.022490	0.0498	0.7615	0.2233
*X*_6_—leaching time (h)	0.043462	2.13908	0.007654	0.0819	0.7050	0.3571
	Regression analysis of the response variables					
Determination coefficient (*R*^2^)	0.9597	0.5861	0.7834			
*p*-value	0.0978	0.7370	0.4567			

*Y*_4_, *Y*_5_, and *Y*_6_ (µg g^−1^) correspond to Au, Ag, and Pd recovery, respectively. Significant effects are underlined

**Table 12:** Regression analysis of the models obtained for the recovery of precious metals using sodium thiosulfate in the second stage

Regression analysis of the independent variables								
	Regression coefficient			*p*-value				
Term	*Y*_4_	*Y*_5_	*Y*_6_	*Y*_4_	*Y*_5_		*Y*_6_	
Constant	0.039425	243.598	0.519726					
*X*_1_—leaching agent concentration (g L^−1^)	0.000872	0.345454	0.001427	0.1891	0.4692		0.2243	
*X*_2_—temperature (°C)	0.001014	− 0.420046	− 0.001309	0.3725	0.6442		0.5091	
*X*_3_—solid/liquid ratio (g L^−1^)	0.000162	− 0.050481	− 0.000020	0.6395	0.8640		0.9742	
*X*_4_—initial pH	0.000876	0.891265	0.004555	0.9305	0.9195		0.8075	
*X*_6_—leaching time (h)	0.003754	− 2.43923	− 0.002165	0.4876	0.5956		0.8167	
	Regression analysis of the response variables							
Determination coefficient (*R*^2^)	0.7549	0.4312	0.6555					
*p*-value	0.5049	0.8779	0.6521					

*Y*_4_, *Y*_5_, and *Y*_6_ (µg g^−1^) correspond to Au, Ag, and Pd recovery, respectively

Based on the results obtained, thiourea was selected as a more efficient leaching agent to recover precious metals, especially Au, in the second stage due to the higher economic value of Au compared to Pd. As approximately the same results were achieved for the recovery of Au in experiments 4 and 5 (0.7520 and 0.7521 µg g^−1^, respectively), the experiment 4 was considered more slightly suitable due to the higher amount of Ag and Pd leached (245.8 and 0.3299 µg g^−1^ compared to 221.3 and 0.3147 µg g^−1^ for experiment 5, respectively) which were obtained under the following conditions: concentration (50 g L^−1^), temperature (30°C), solid/liquid ratio (90 g L^−1^), initial pH (1), and leaching time (6 h). However, in order to determine the optimal leaching agent and conditions that can be applied on an industrial scale, it is necessary to carry out the optimization and consider the economic evaluation of the process such as operational costs, leaching agent and energy consumption, and the price of precious metals.

## Conclusions

A hydrometallurgical process was proposed for the leaching of valuable metals from waste motherboards’ PCBs using thiourea and sodium thiosulfate in an environmentally friendly manner. The results obtained demonstrated that the quantities of metals leached using thiourea and sodium thiosulfate in a single-stage process are relatively low, especially in the case of precious metals, which can limit its application. The results obtained for the sequential recovery of base and precious metals from PCBs indicated that nitric acid is capable of leaching much higher amounts of Cu (the predominant metal in PCBs) and Pb (environmentally problematic) than hydrochloric acid in the first stage, whereas hydrochloric acid was found to leach much more Sn. Screening design showed that HNO_3_ concentration, temperature, average particle size, and leaching time are significant parameters on the leaching of Cu and Pb and limit that of Au, Ag, and Pd. The optimal conditions obtained from the Definitive Screening Design using HNO_3_ to maximize the leaching of Cu and Pb and minimize the extraction of Au, Ag, and Pd in the range studied were achieved to be 3.4 M, 35 °C, solid/liquid ratio of 90 g L^−1^, and particle size between 1 and 2 mm for 2 h of leaching under constant stirring rate managing to leach amounts of 355,955 and 19,250 µg g^−1^ for Cu and Pb, respectively. The acid leaching stage of base metals had favorable effects on the subsequent leaching of precious metals (Au, Ag, and Pd) using thiourea and sodium thiosulfate, significantly improving the leached amounts compared to the one-stage process. Particularly, the amount of Ag leached increased from 2.55 to 245.8 µg g^−1^ for thiourea and from 2.42 to 258.0 µg g^−1^ for sodium thiosulfate. Initial pH was the only factor influencing the leaching of precious metals, affecting Au removal with thiourea, in the second stage. Thiourea was found to be more appropriate than sodium thiosulfate for the recovery of precious metals, particularly owing to the greater Au leaching. The more suitable conditions using thiourea were considered to be 50 g L^−1^, 30°C, solid/liquid ratio of 90 g L^−1^, and initial pH of 1 for 6 h according to the ultimate recovery of precious metals. This study was mainly focused on screening the variables that can affect the selective leaching of base and precious metals from waste motherboards’ PCBs. However, for future work, the possibility of adding an oxidant together with thiourea to improve the leaching of precious metals in the second stage will be studied considering the economic assessment of the process.

## Supplementary Information

Below is the link to the electronic supplementary material.

Supplementary file1 (DOCX 1.94 MB)

## Data Availability

All data generated or analyzed during this study are included in this article.
