# Supplementary material for: Screening of variables affecting the selective leaching of valuable metals from waste motherboards’ PCBs
Source: Environ Sci Pollut Res Int. 2024 Mar 9;32(48):27651–66. doi: 10.1007/s11356-024-32793-1 (PMC12695914; doi:10.1007/s11356-024-32793-1)
Supplement: Supplementary file 1 — Supplementary file1 (DOCX 1.94 MB) [file 11356_2024_32793_MOESM1_ESM.docx]

**Supplementary materials**

Screening of variables affecting the selective leaching of valuable metals from waste motherboards’ PCBs

Vahid Rahimi · Emilio Antonio Inzulza-Moraga · Diego Gómez-Díaz · María Sonia Freire · Julia González-Álvarez^*^

Department of Chemical Engineering, School of Engineering, Universidade de Santiago de Compostela, Rúa Lope Gómez de Marzoa s/n, 15782, Santiago de Compostela, Spain

^*^ Corresponding author e-mail address: julia.gonzalez@usc.es

^*^ Corresponding author phone number: (+34) 881816761

**List of Tables:**

**Table S1** Regression analysis of the models obtained for the recovery of metals using thiourea in a single-stage process

| Regression analysis of the independent variables | | | | | | | | | | | |
| --- | --- | --- | --- | --- | --- | --- | --- | --- | --- | --- | --- |
|  | Regression coefficient | | | | |  | *p*-value | | | | |
| Term | **Y_1_** | **Y_2_** | **Y_3_** | **Y_5_** | **Y_6_** |  | **Y_1_** | **Y_2_** | **Y_3_** | **Y_5_** | **Y_6_** |
| Constant | 12822.7 | 2331.31 | 1432.03 | 0.056567 | 0.182309 |  |  |  |  |  |  |
| X_1_ - thiourea concentration (g L^-1^) | 644.296 | - 15.2498 | - 9.42967 | 0.011045 | 0.000005 |  | 0.0728 | 0.0591 | 0.0650 | 0.3183 | 0.9947 |
| X_2_ - temperature (°C) | - 89.1978 | - 1.15468 | - 0.249737 | 0.007822 | - 0.001008 |  | 0.7664 | 0.8610 | 0.9527 | 0.4682 | 0.2453 |
| X_3_ - solid/liquid ratio (g L^-1^) | - 139.606 | - 4.35578 | - 3.5413 | - 0.002013 | - 0.00060 |  | 0.2743 | 0.1427 | 0.0779 | 0.6350 | 0.1075 |
| X_4_ - initial pH | - 9242.45 | - 164.538 | - 95.5879 | - 0.228404 | - 0.021216 |  | 0.0227 | 0.0467 | 0.0625 | 0.0705 | 0.0393 |
| X_5_- average particle size (mm) | 15066.7 | - 346.387 | - 298.294 | 0.210611 | 0.000254 |  | 0.1728 | 0.1581 | 0.0756 | 0.5540 | 0.9925 |
| X_6_ - leaching time (h) | 3375.84 | - 36.7859 | - 5.46936 | 0.084949 | 0.003434 |  | 0.1350 | 0.4188 | 0.8456 | 0.2572 | 0.5310 |
| Regression analysis of the response variables | | | | | | | | | | | |
| Determination coefficient (R^2^) | 0.8215 | 0.7951 | 0.8092 | 0.6524 | 0.7325 |  |  |  |  |  |  |
| *p*-value | 0.0809 | 0.1093 | 0.0936 | 0.3200 | 0.1916 |  |  |  |  |  |  |
| Y_1_, Y_2_, Y_3_, Y_5_ and Y_6_ (µg g^-1^) correspond to Cu, Pb, Sn, Ag and Pd recovery, respectively. | | | | | | | | | | | |

**Table S2** Regression analysis of the models obtained for the recovery of metals using sodium thiosulfate in a single-stage process

|  | Regression analysis of the independent variables | | | | | | | | | | | | |  |
| --- | --- | --- | --- | --- | --- | --- | --- | --- | --- | --- | --- | --- | --- | --- |
|  | | Regression coefficient | | | | |  |  | *p*-value | | | | |  |
| Term | | **Y_1_** | **Y_2_** | **Y_3_** | **Y_4_** | **Y_5_** | Y_6_ |  | **Y_1_** | **Y_2_** | **Y_3_** | **Y_4_** | **Y_5_** | Y_6_ |
| Constant | | 14415.1 | - 7.98919 | - 38.4872 | 0.129774 | - 0.472825 | - 0.019153 |  |  |  |  |  |  |  |
| X_1_ - thiosulfate concentration (g L^-1^) | | 100.388 | - 0.033288 | 0.549467 | - 0.000079 | 0.016462 | - 0.000157 |  | 0.2287 | 0.5189 | 0.0452 | 0.7532 | 0.0912 | 0.7157 |
| X_2_ - temperature (°C) | | 126.969 | 0.064628 | 0.499183 | - 0.001183 | 0.006470 | - 0.001005 |  | 0.1435 | 0.2360 | 0.0607 | 0.0043 | 0.4492 | 0.0571 |
| X_3_ - solid/liquid ratio (g L^-1^) | | - 68.2735 | 0.014015 | - 0.127299 | - 0.000256 | - 0.005212 | - 0.000087 |  | 0.0671 | 0.4981 | 0.1848 | 0.0438 | 0.1593 | 0.6168 |
| X_4_ - initial pH | | - 1064.84 | 0.626248 | 3.20139 | - 0.003354 | 0.130904 | 0.010987 |  | 0.2056 | 0.2488 | 0.1826 | 0.2188 | 0.1578 | 0.0433 |
| X_5_- average particle size (mm) | | - 578.464 | - 1.72202 | 9.86827 | 0.002935 | - 0.100379 | 0.018437 |  | 0.8221 | 0.3309 | 0.2120 | 0.7272 | 0.7182 | 0.2337 |
| X_6_ - leaching time (h) | | 314.711 | 0.238789 | - 2.13265 | 0.000377 | - 0.057155 | - 0.001823 |  | 0.5475 | 0.4891 | 0.1829 | 0.8222 | 0.3265 | 0.5329 |
|  | Regression analysis of the response variables | | | | | | | | | | | | |  |
| Determination coefficient (R^2^) | | 0.7208 | 0.5554 | 0.8152 | 0.8720 | 0.7032 | 0.7620 |  |  |  |  |  |  |  |
| *p*-value | | 0.2091 | 0.4928 | 0.0873 | 0.0382 | 0.2362 | 0.1505 |  |  |  |  |  |  |  |
| Y_1_, Y_2_, Y_3_, Y_4_, Y_5_ and Y_6_ (µg g^-1^) correspond to Cu, Pb, Sn, Au, Ag and Pd recovery, respectively. | | | | | | | | | | | | | | |

**List of Figures:**

1. b) c) d)


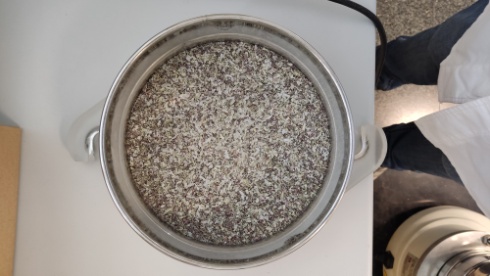

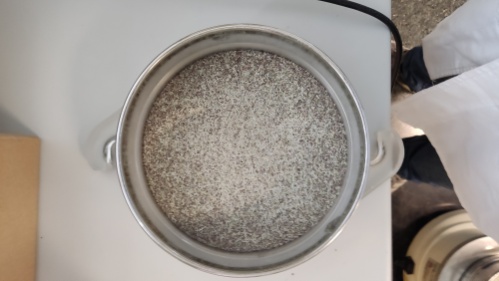

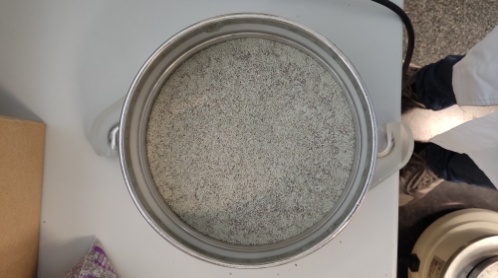

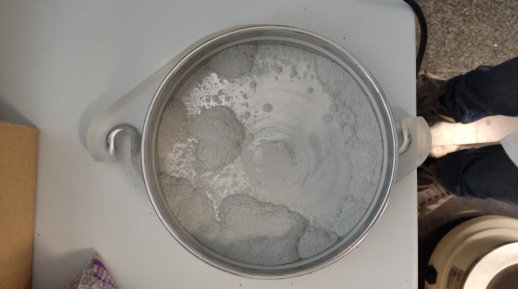


**Fig. S1** Particle size ranges after sieving: (a) 1-2 mm (b) 0.5-1 mm (c) 0.1-0.5 mm and (d) ˂ 0.1 mm


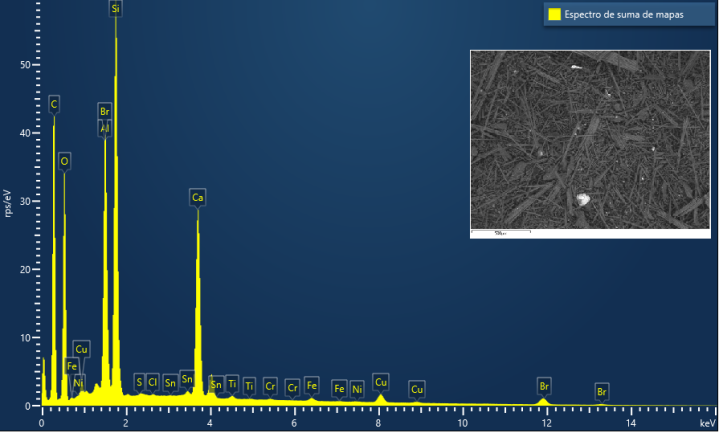

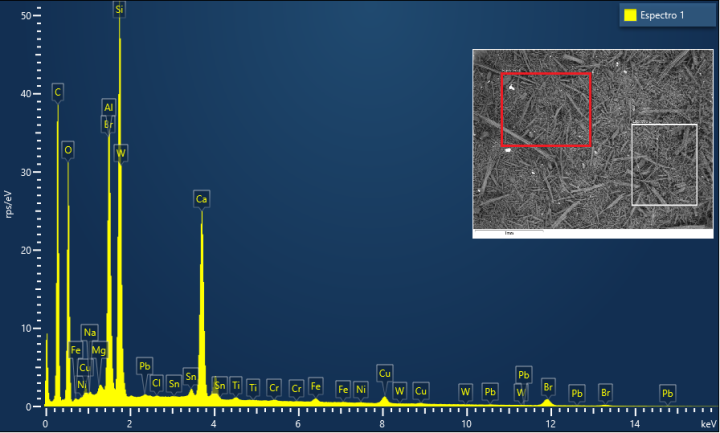


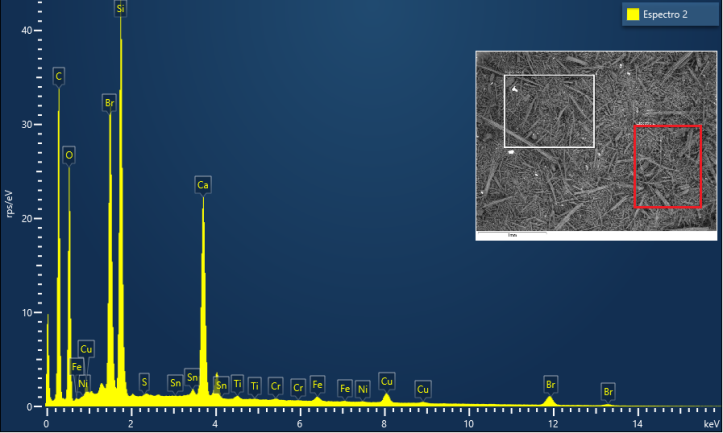


**Fig. S2** EDX spectra corresponding to particle size lower than 0.1 mm obtained from different specific areas of PCB surfaces.


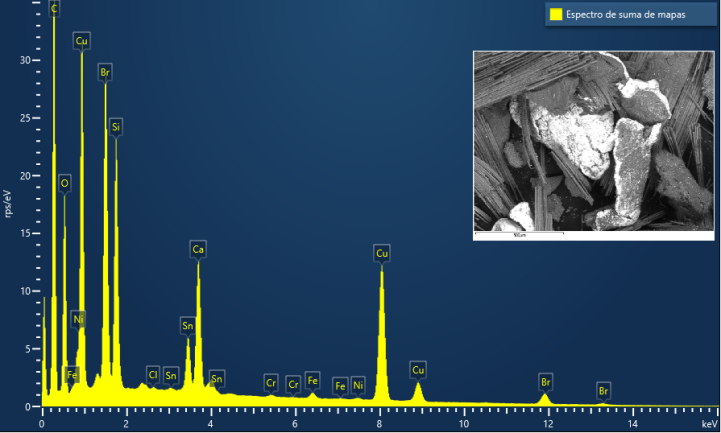

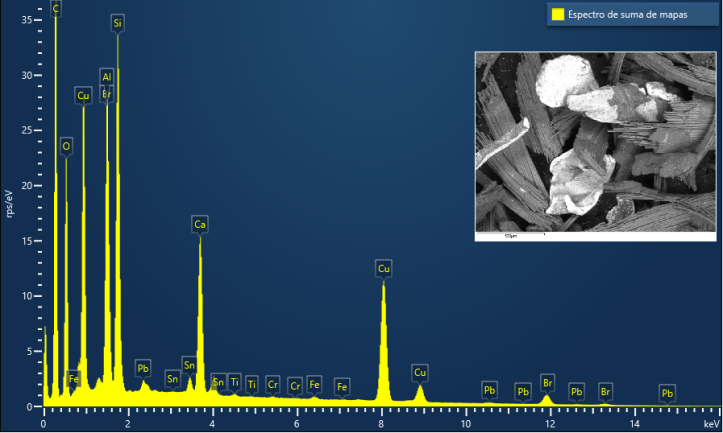


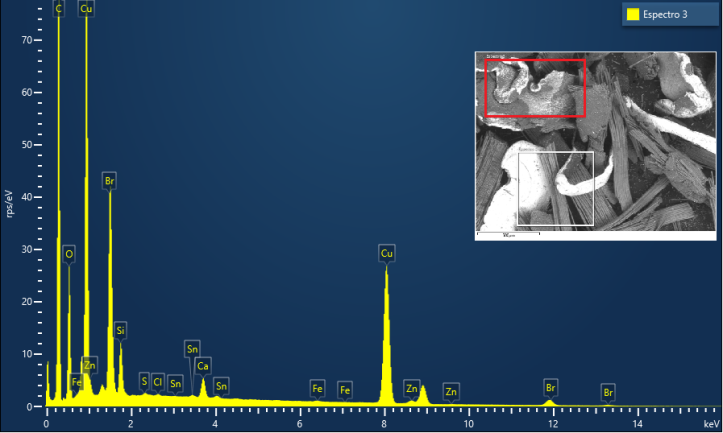

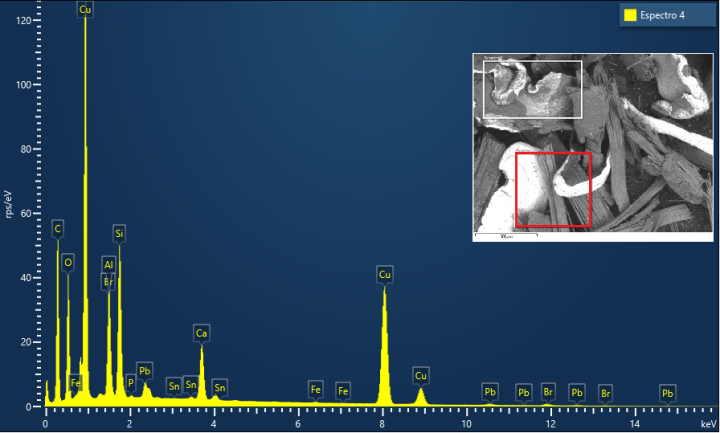


**Fig. S3** EDX spectra corresponding to particle size 0.1-0.5 mm obtained from different specific areas of PCB surfaces.


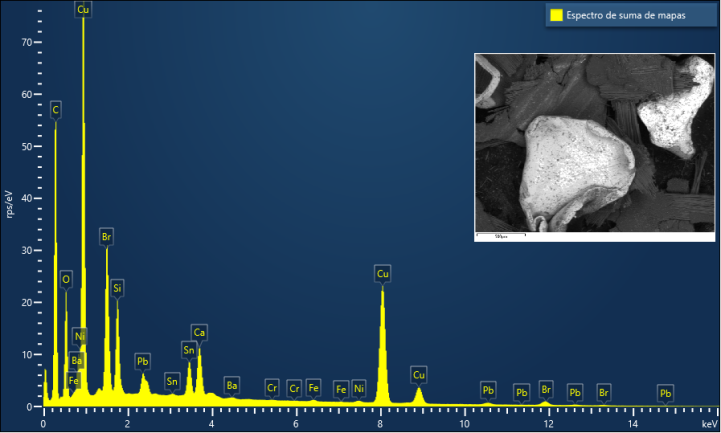

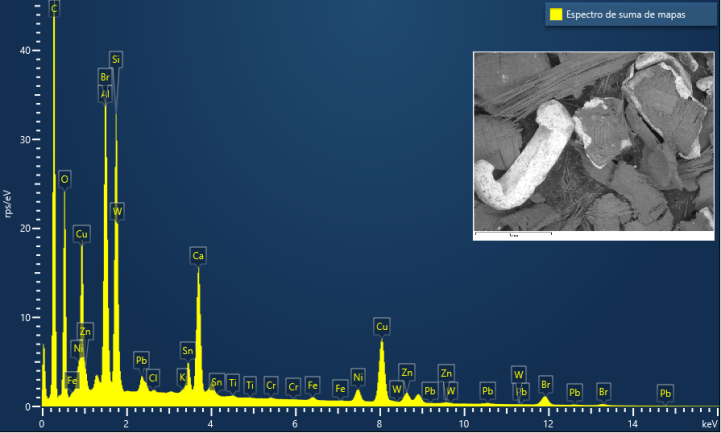


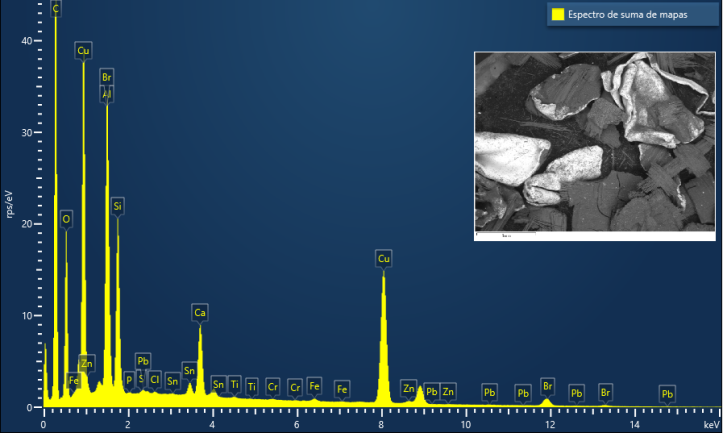


**Fig. S4** EDX spectra corresponding to particle size 0.5-1.0 mm obtained from different specific areas of PCB surfaces.


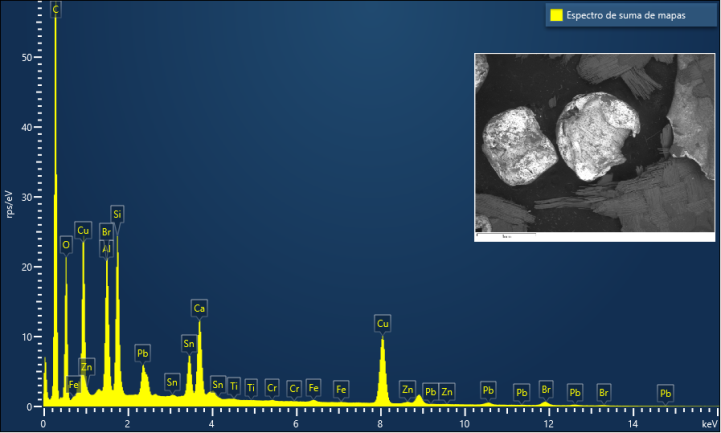

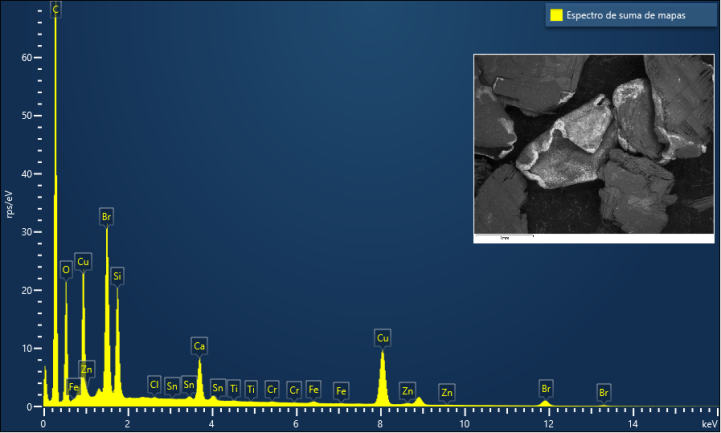


**Fig. S5** EDX spectra corresponding to particle size 1.0-2.0 mm obtained from different specific areas of PCB surfaces.


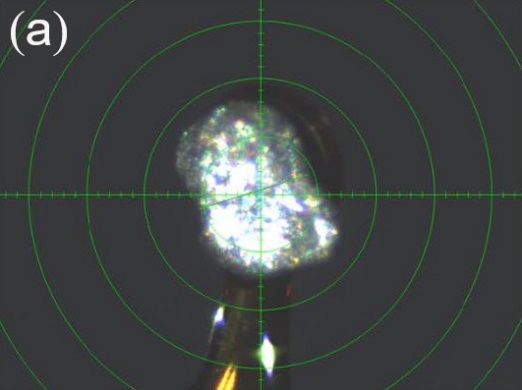

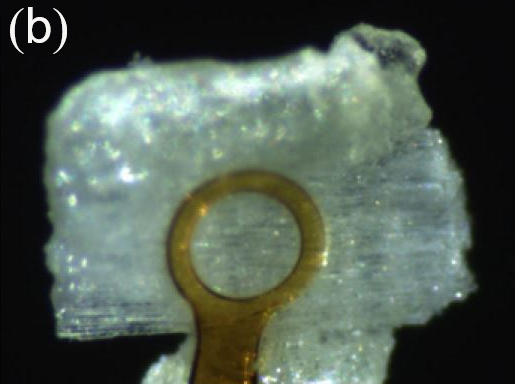

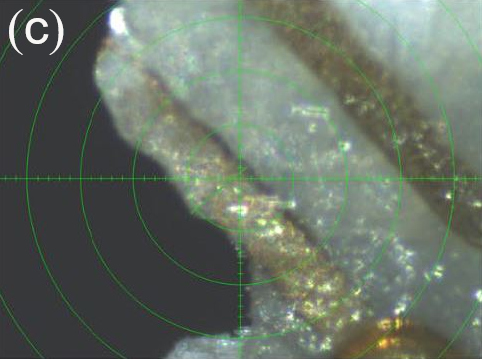


**Fig. S6** 2D images for determination of µ-XRD diffractograms corresponding to different particle size ranges: (a) 0.1-0.5 mm (b) 0.5-1.0 mm and (c) 1.0-2.0 mm

**Fig. S7** Observed versus predicted values by the Definitive Screening Design for the recovery of: (a) Cu (b) Pb (c) Au (d) Ag and (e) Pd
